# Retrospective analysis of perineal herniorrhaphy with cone-shaped polypropylene mesh in dogs: technique description and outcome

**DOI:** 10.3389/fvets.2023.1279776

**Published:** 2023-10-26

**Authors:** Tatsuya Heishima, Kumiko Ishigaki, Mamiko Seki, Kenji Teshima, Orie Yoshida, Kaito Iida, Ryo Takeuchi, Kazushi Asano

**Affiliations:** Laboratory of Veterinary Surgery, Department of Veterinary Medicine, College of Bioresource Science, Nihon University, Fujisawa, Kanagawa, Japan

**Keywords:** dog, perineal hernia, polypropylene mesh, reconstruction, surgery

## Abstract

**Introduction:**

This study aimed to describe the technique for the surgical repair of perineal hernia (PH) in dogs using a polypropylene mesh (PM) and to evaluate its outcomes.

**Methods:**

All dogs were placed in the Trendelenburg position. Castration and caudal celiotomy for cystopexy and colopexy were performed as needed. Ipsilateral perianal incision was performed in the affected hernia side. A PM was formed a cone-shape with suturing and placed in hernial foramen. The PH was repaired with suturing between PH and pelvic diaphragm including the sacrotuberous ligament, internal obturator muscle, and external anal sphincter muscle. The medical records of all dogs were reviewed to evaluate signalment, perioperative findings, postoperative complications, and prognosis.

**Results:**

Of the 22 dogs, 15 were intact, and 7 were previously neutered. The median age and body weight were 10 years and 6.8 kg, respectively. The PH reconstruction using a cone-shaped PM was feasible in all dogs. The median operative time was 60.5 min for unilateral PH and 109 min for bilateral PH. Major postoperative complications occurred in seven dogs (32%), and three dogs (14%) had a recurrence of PH. In the long-term (> 2 weeks) follow-up period, 16 dogs (73%) had an excellent prognosis.

**Discussion:**

Our study suggests that PH reconstruction surgery using a cone-shaped PM may be a viable treatment method for PH in dogs. Therefore, a cone-shaped PM could serve as an alternative treatment option for canine PH reconstruction.

## 1. Introduction

In dogs, perineal hernia (PH) is a condition caused by the prolapse of organs in the pelvic and abdominal cavities into the subcutaneous tissue of the perineum due to atrophy of the levator ani and coccygeus muscles, which comprise the pelvic diaphragm. PH is most commonly observed in middle-aged to elderly male dogs, and almost all affected dogs are intact male dogs (1).

Surgery is the treatment of choice for PH in dogs (1–4). Autologous transplant techniques using tissues, such as the internal obturator muscle (5, 6), semitendinosus muscle (7), fascia lata (8), sacrotuberous ligament (9), and tunica vaginalis communis (10–12), are now being developed and refined for PH with severe muscle atrophy reconstructive surgery. Previous studies have reported that 51.2–93% of cases had no postoperative complications for more than 1 year after internal obturator transposition (IOT) (5, 6). The recurrence rate after IOT surgery was reported to be 0–27.8% (5, 6). In recurrent cases after IOT surgery or in case with the atrophy of internal obturator muscles, IOT surgery has the potential risk for the failure of the PH reconstruction.

Some surgical techniques use artificial materials to repair canine PH. Artificial materials may be indicated even when reconstruction with autologous tissue is difficult, such as in cases of severe muscle atrophy or postoperative recurrence. Of the artificial materials, polypropylene mesh (PM) is pliable, very strong, and unaffected by exposure to water (13). There are some reports on PH reconstructive surgery using PM; a sheet-shaped PM has been commonly applied to cover the hernial foramen following IOT (14–16). It should be possible to use PM for the repair of PH without the combination of IOT; however, there is limited information on PH reconstruction using PM only. We hypothesized that implantation of the PM, which forms in a cone shape into the hernial defect, would result in only easier and safer PH reconstruction. Therefore, the purpose of this study was to describe the surgical procedure for herniorrhaphy using a cone-shaped PM for PH in dogs and evaluate the outcomes and complications.

## 2. Methods

### 2.1. Animals

Twenty-two dogs diagnosed with PH were referred to our hospital. In the initial evaluation, all dogs had the clinical symptoms of PH including tenesmus and swelling of unilateral/bilateral perineal areas. In addition, digital rectal examination and abdominal radiography were used to diagnose PH. Complete blood count (CBC) and serum chemistry were performed to rule out other diseases and as pre-surgical/anesthetic tests. All dogs underwent perineal herniorrhaphy with a cone-shaped PM. Prior to the first evaluation, informed consent was obtained from all owners for all procedures. All tests and procedures were approved by our institutional review board in compliance with the ARRIVE guidelines.

### 2.2. General anesthesia and perioperative management

For premedication, 1.0 mg/kg maropitant citrate hydrate (Cerenia^®^; Zoetis, Parsippany, NJ, United States) and 0.04 mg/kg atropine sulfate (Nipro Co., Settsu, Japan) were administered subcutaneously. In addition, 5.0 μg/kg fentanyl (Terumo Co., Tokyo, Japan) were commonly administered intravenously (IV). Depending on the patient’s condition, 0.1 mg/kg midazolam (Dormicum^®^; Astellas Pharma Inc., Tokyo, Japan) was added or replaced the IV administration of 5.0 μg/kg fentanyl and 0.25 mg/kg droperidol (Thalamonal^®^; Daiichi-Sankyo ProPharma Co., Ltd., Tokyo, Japan) (0.1 mL/kg). General anesthesia was induced by IV administration of propofol (Mylan; Mylan Seiyaku Ltd., Tokyo, Japan), and endotracheal intubation was performed. During surgery, anesthesia was maintained with isoflurane (IsoFlo^®^; Zoetis), and artificial ventilation was carried out using oxygen.

Continuous rate infusion (CRI) of 10–40 μg/kg/h remifentanil hydrochloride (Daiichi-Sankyo ProPharma Co., Ltd.) was also performed for intraoperative analgesia. Additional CRI of 25–50 μg/kg/min lidocaine (Aspen Japan, Tokyo, Japan) was carried out in some cases. As an extra additional analgesic treatment, a bolus dose of 0.3 mg/kg morphine hydrochloride (Takeda Pharmaceutical Co., Ltd., Osaka, Japan) was administered intramuscularly before and after surgery as needed. Postoperatively, CRI of 1.25–5.0 μg/kg/h remifentanil hydrochloride (Daiichi-Sankyo ProPharma Co., Ltd.) or 2.5–5.0 μg/kg fentanyl (Terumo Co.) was used for postoperative analgesia.

### 2.3. Surgical procedure

**Figure 1:**
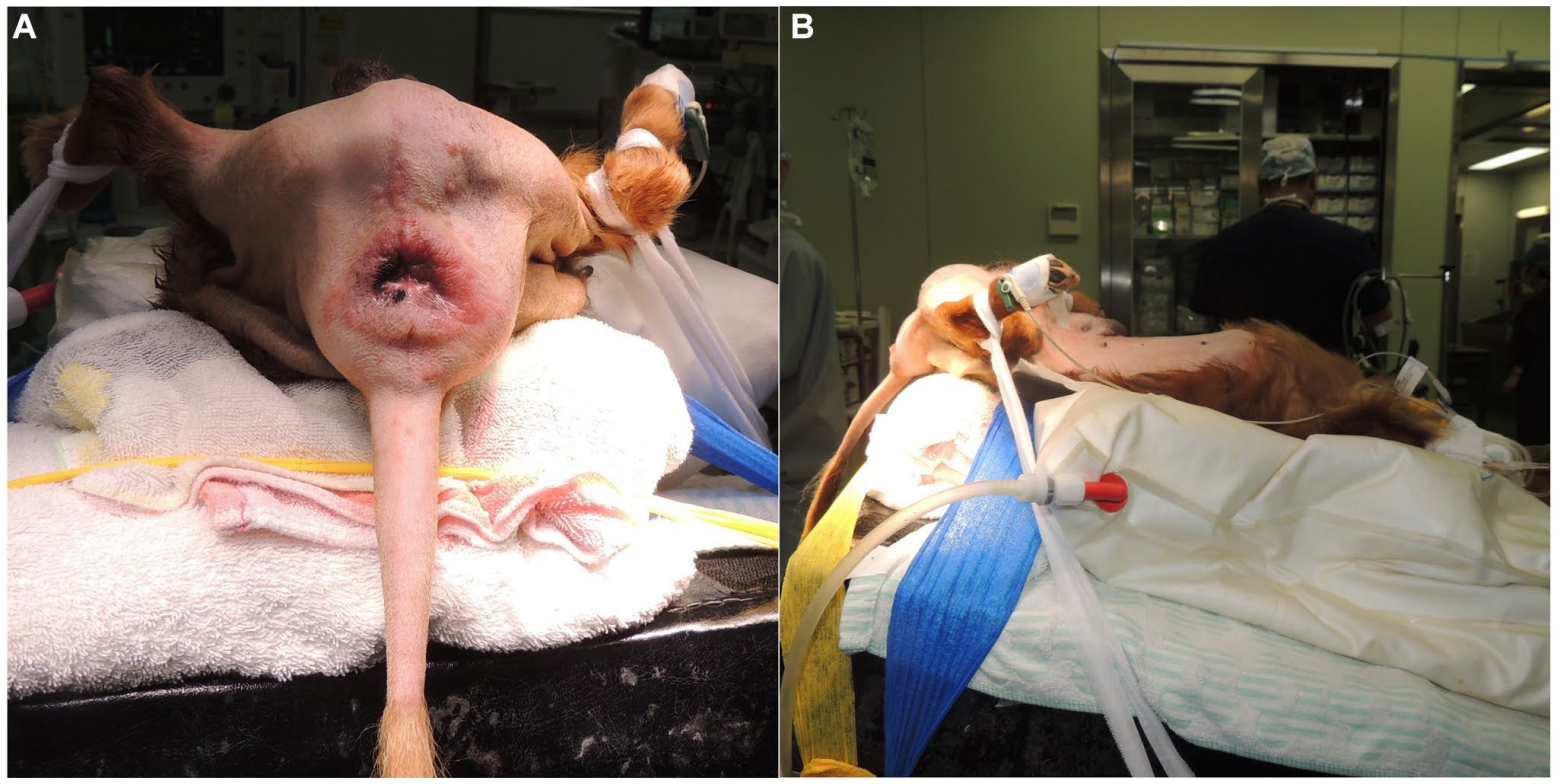
The canine patients were positioned for the reconstruction of the perineal hernia with a cone-shaped polypropylene mesh. The canine patients were secured at the edge of the operating table in the Trendelenburg position, with the hind legs pulled sideways and the tail hanging down. The photographs were taken from the caudal side **(A)** and the left side **(B)**.

After preparing for aseptic surgery, all dogs were placed in the Trendelenburg position at the edge of the operating table, with the hind limbs pulled sideways and the tail hanging down (Figure 1), as previously described (12).

**Figure 2:**
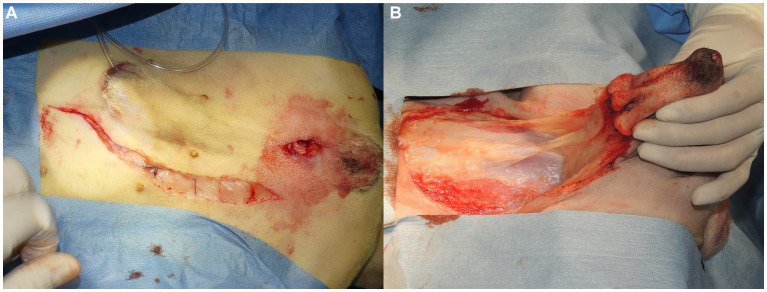
**(A)** When colopexy or cystopexy was performed, a skin incision was made on the left-hand side of the penis to make a caudal abdominal midline incision. **(B)** It was difficult to reposition anatomically displaced organs in severe cases where pelvic organs, such as the colon and bladder, were not only caudally displaced, but had also prolapsed into the perineal area. Thus, an inverted U-shaped skin incision was made around the foreskin to secure a wide surgical field, the penis was everted with blunt dissection of the subcutaneous tissue, and caudal laparotomy was performed with an incision from the umbilicus to the pubis.

In intact male dogs, prescrotal castration was routinely performed on both sides. An abdominal midline skin incision was curved to the right side of the penis and prepuce and extended to the level of the pubis (Figure 2A), when the displacement of the bladder and colon into the perineum was severe. In some cases, it was necessary to ensure a wide surgical field, a preputial U-shaped skin incision was made around the foreskin (Figure 2B), as previously described (17). After caudal celiotomy, colopexy was performed when the caudal displacement of the anus occurred due to severe fecal retention based on clinical signs and abdominal lateral radiography. In addition, cystopexy was performed when the caudal displacement of the bladder and prostate was observed on lateral abdominal radiography. The colon and bladder were sutured and fixed to the left and right abdominal walls, respectively, with a 3–0 polydioxanone suture (PDS II®; Johnson & Johnson, New Brunswick, NJ, United States), as needed.

**Figure 3:**
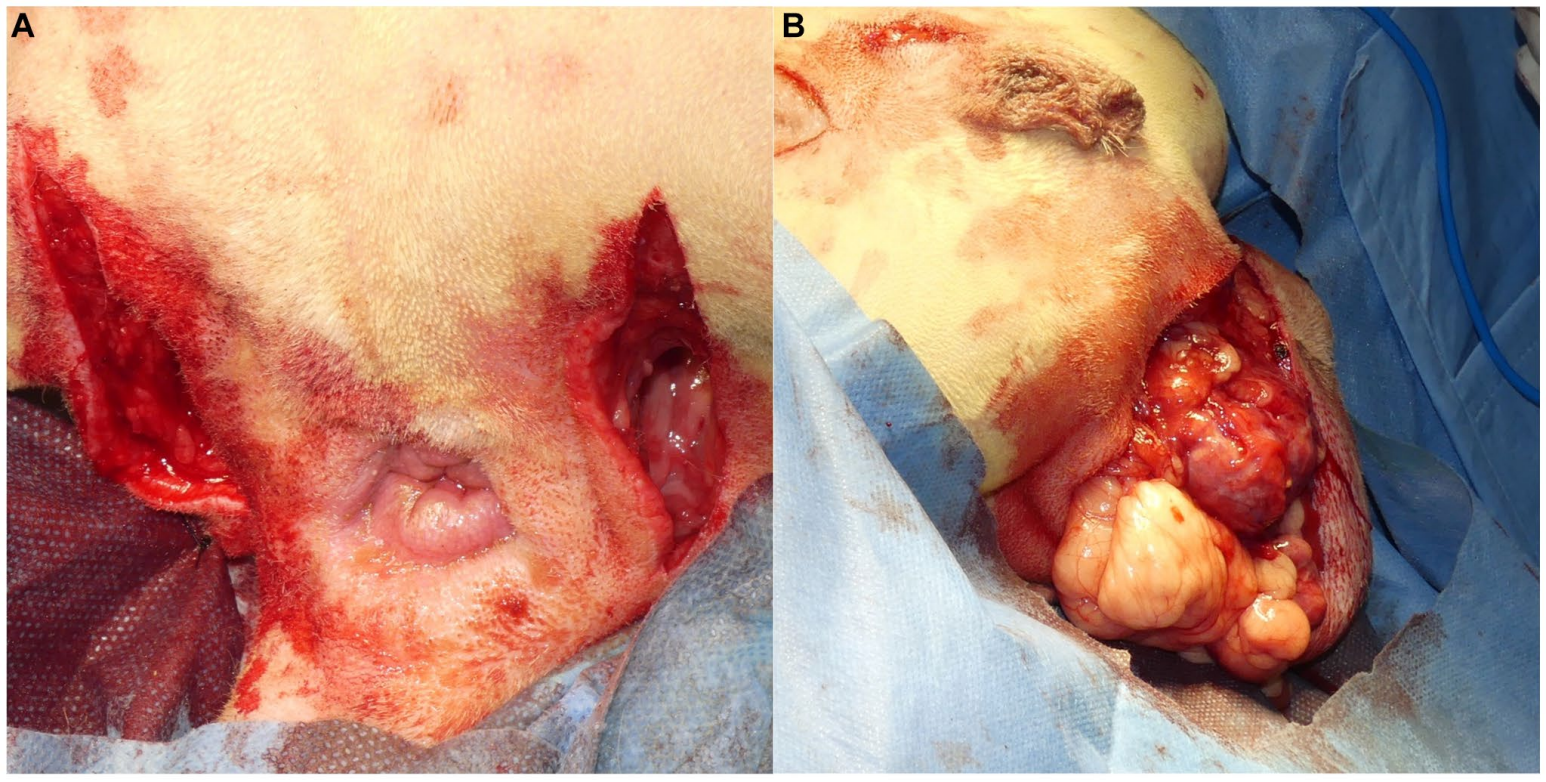
**(A)** An incision was made in the perineal area from the ischial tuberosity on the affected side to the lateral side of the anus, close to the base of the tail. **(B)** In cases with bilateral perineal hernias and severe prolapse of the organs into the perineal area, where it was necessary to secure the surgical field on the ventral side of the anus, a semicircular incision was made on the ventral side of the anus to facilitate confirmation of the position of organs prolapsed into the perineal area and the anatomical findings of the pelvic septum. Furthermore, making a semicircular incision facilitates suturing after trimming when the hernia sac is large, necessitating significant trimming of the skin.

After placing the patient in the Trendelenburg position, a skin incision was made over the affected part of the PH on one or both sides (Figure 3A). In the case of a ventral hernia, in addition to the bilateral PH, a semicircular incision at the ventral side of the anus was made on the perineum (Figure 3B). Subcutaneous tissues were dissected to confirm PH. The anatomical positions and atrophy of the pelvic septal structures, including the sacrotuberous ligament, internal obturator muscle, ischium and external anal sphincter muscle, were confirmed visually and through digital palpation.

**Figure 4:**
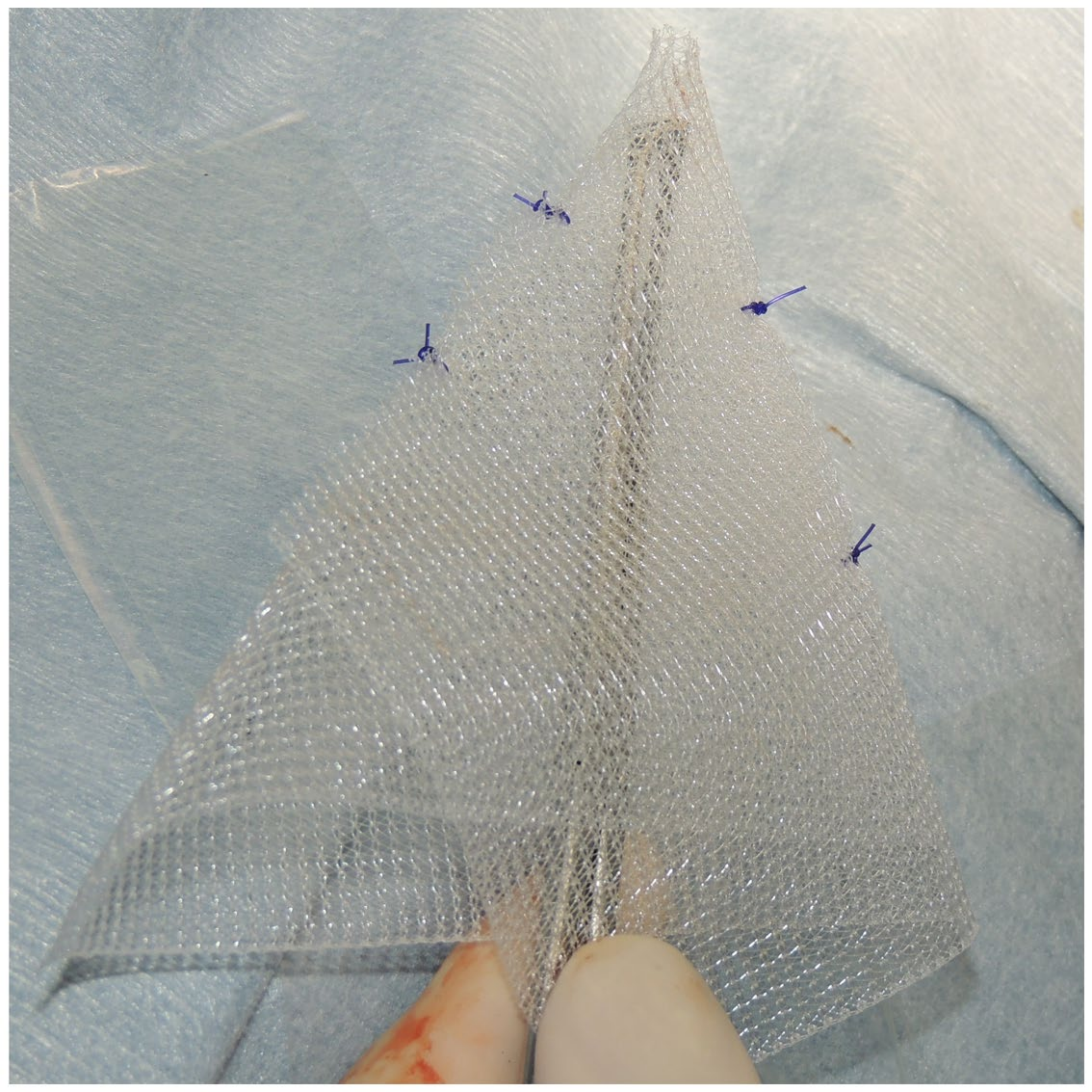
The mesh used for reconstruction of the perineal hernias was polypropylene mesh (PROLENE^®^; Johnson & Johnson), measuring 15 cm × 15 cm. The mesh was folded and shaped into a cone to match the hernial defect. The cone-shaped mesh was sutured and secured in two to three places with 2–0 polypropylene sutures (PROLENE^®^; Johnson & Johnson). Finally, the cone-shaped mesh was trimmed to fit the size of the dog.

**Figure 5:**
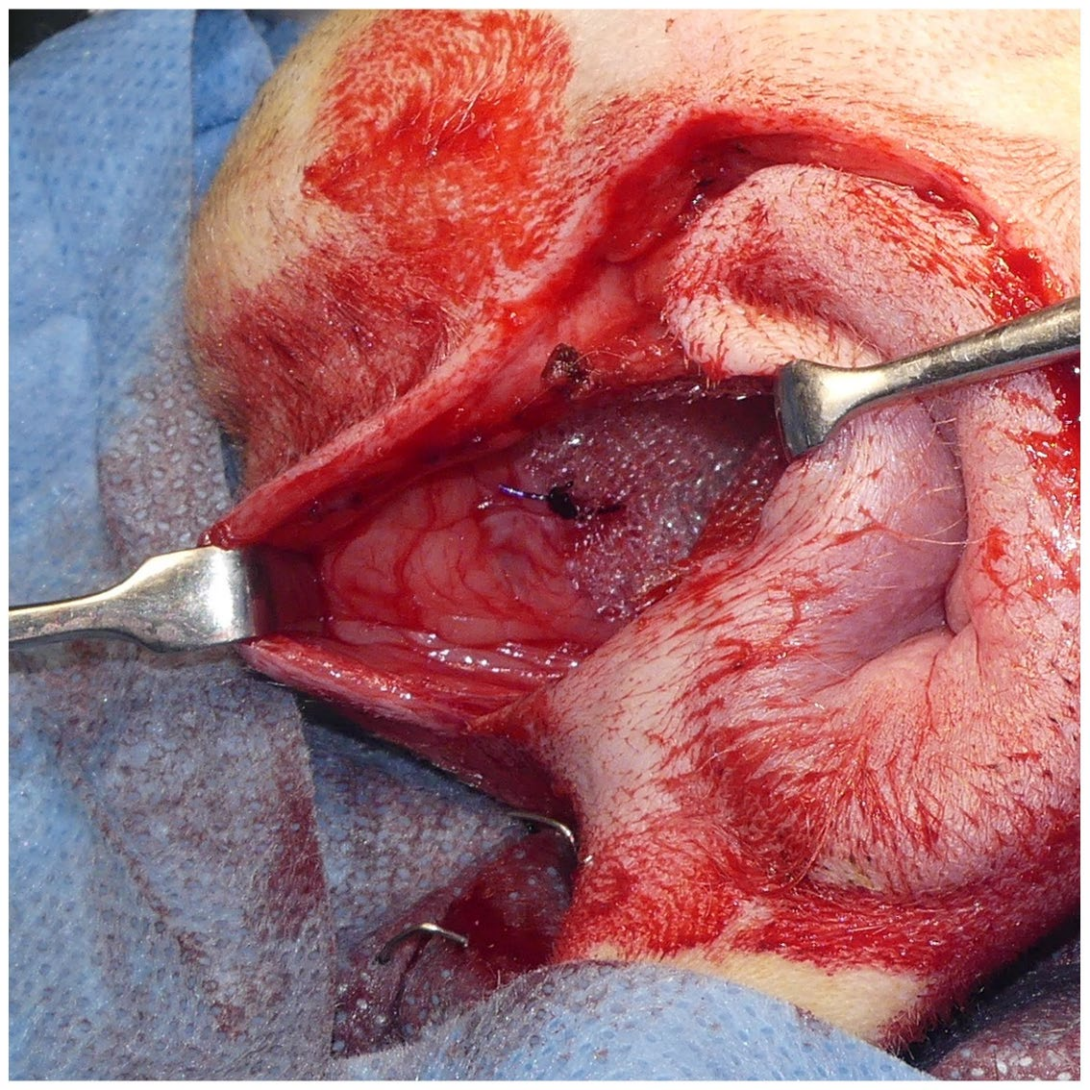
The cone-shaped mesh was placed in the hernial foramen. The lateral side of the mesh was sutured to the sacrotuberous ligament with 2–0 polypropylene sutures (PROLENE^®^; Johnson & Johnson). The sciatic nerve is located adjacent to the sacrotuberous ligament; thus, care was taken to avoid injuring the sciatic nerve during suturing.

**Figure 6:**
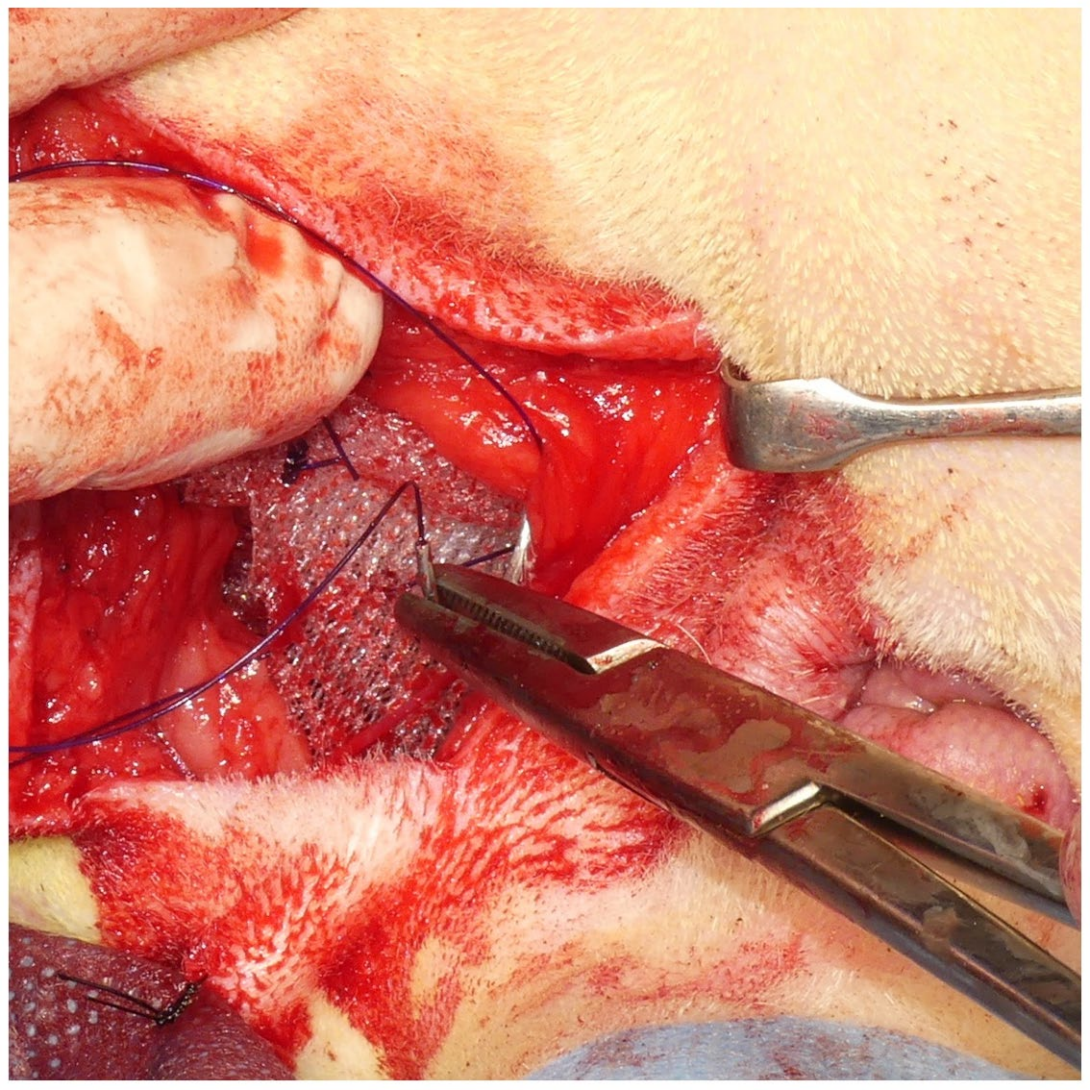
After suturing the cone-shaped mesh to the sacrotuberous ligament, the ventral side of the mesh was sutured to the internal obturator muscle. When the internal obturator muscle was atrophied, the sciatic periosteum was sutured together with the internal obturator muscle.

**Figure 7:**
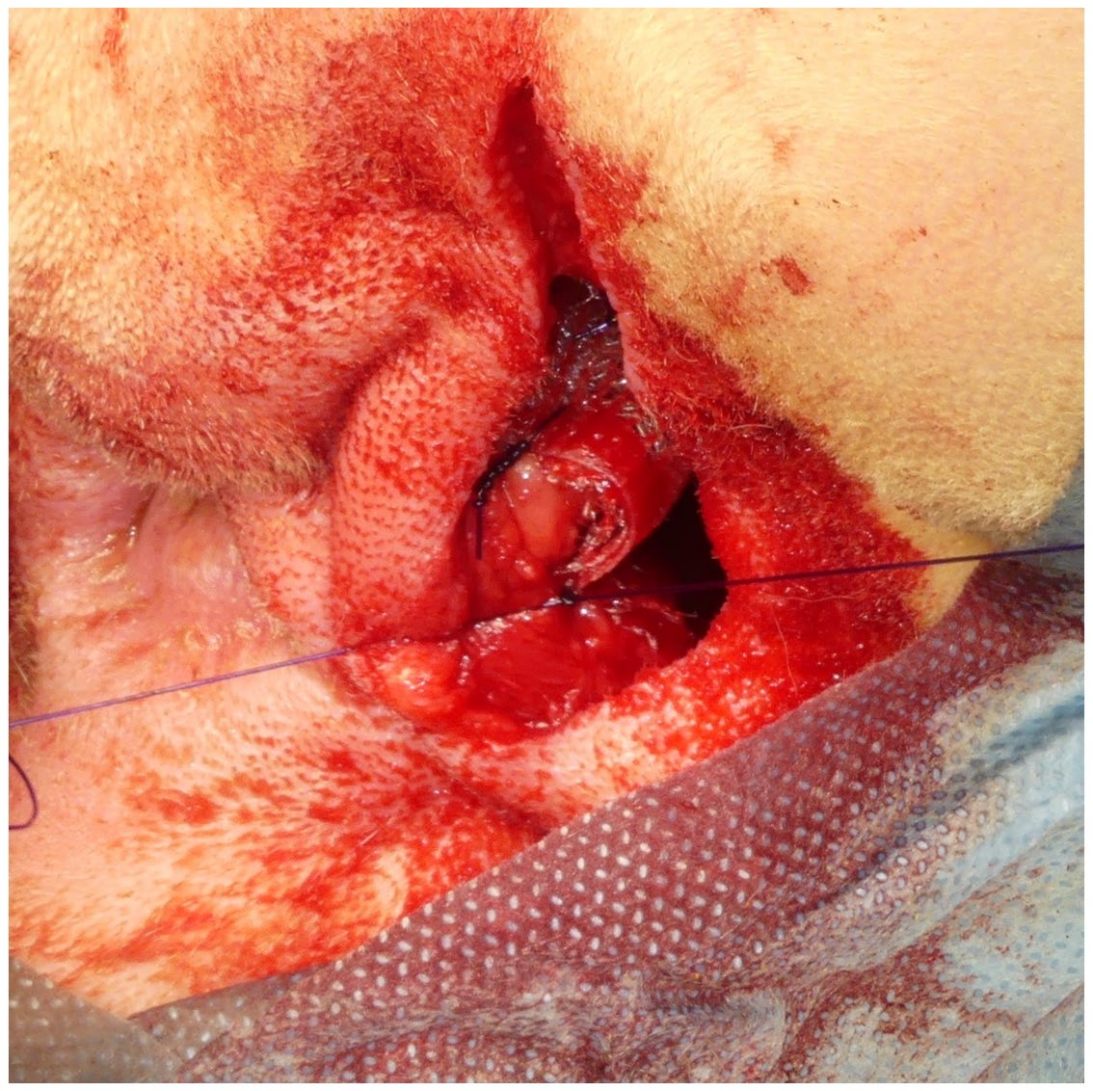
The medial side of the cone-shaped mesh was sutured to the external anal sphincter. The surgeon inserted a finger into the rectum and sutured the mesh to the external anal sphincter around the finger.

**Figure 8:**
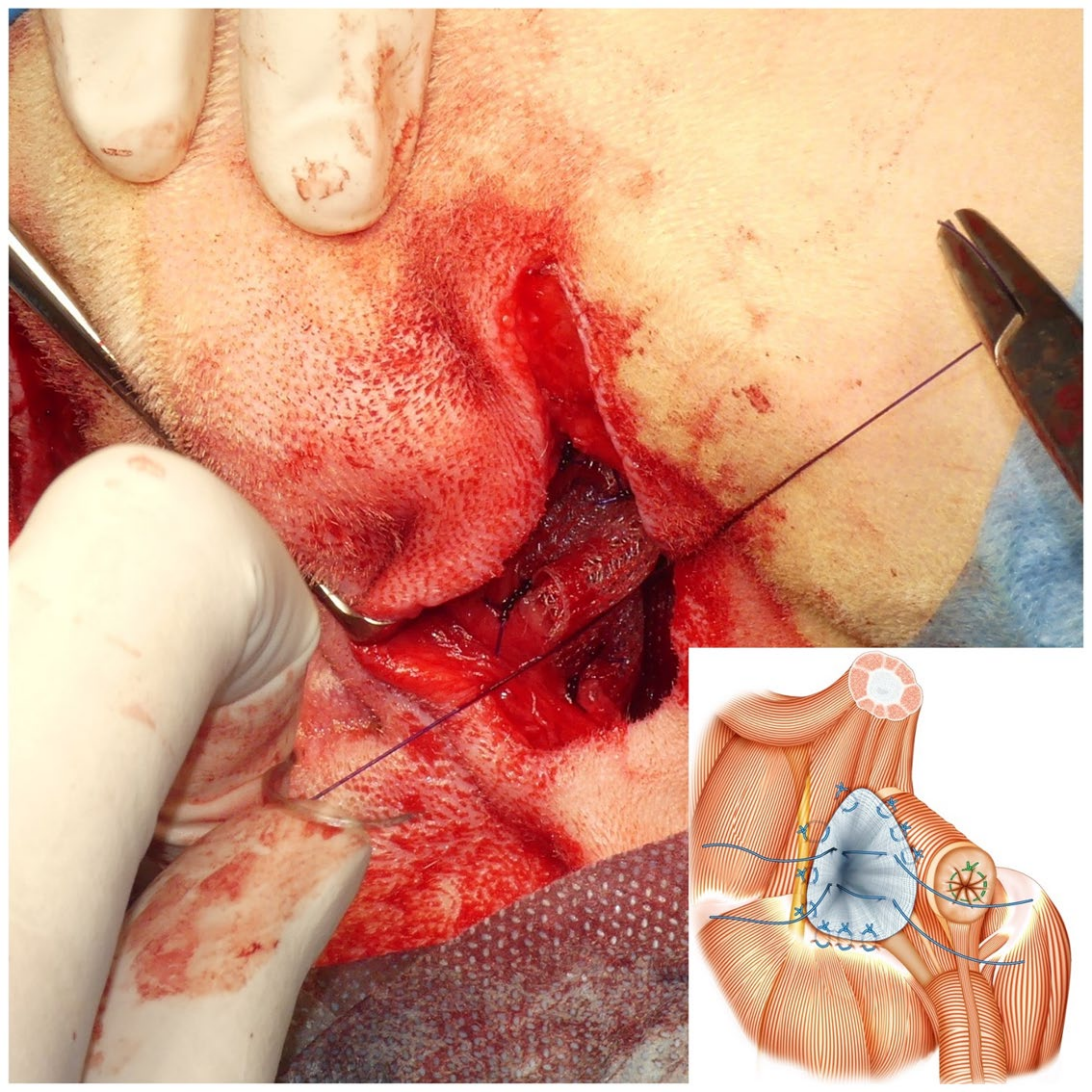
After suturing the cone-shaped mesh to the pelvic septum, the medial surfaces of the mesh were sutured together to pull the anus cranially, thereby reducing the space of the hernial foramen. The schematic diagram shows how the sutures were threaded through the external anal sphincter side and sacrotuberous ligament side of the mesh after suturing the mesh to the sacrotuberous ligament, internal obturator muscle, and external anal sphincter.

A square PM (PROLENE^®^; Johnson & Johnson) of 15 cm in length was folded to form a cone shape with sutures (Figure 4). The pointed end of the cone-shaped PM was inserted into the hernial defect, and its size was verified. First, the PM was fixed to the sacrotuberous ligament using 3–4 sutures (Figure 5). Subsequently, the PM was fixed to the internal obturator muscle and ischial periosteum using 3–4 sutures (Figure 6). The PM was fixed to the external anal sphincter and the remaining coccygeal muscles with 3–4 sutures (Figure 7). Finally, the PM was sutured to itself on the sacrotuberous ligament and external anal sphincter sides (Figure 8) to restore the anus in its original position. For all sutures, 2–0 polydioxanone (PDSII^®^; Johnson & Johnson) with a taperpoint needle of 5/8 circle body was used. Following the completion of PH reconstruction, the subcutaneous tissues and skin were routinely closed. In cases where colopexy, cystopexy, or both were carried out, the abdominal incision was routinely closed.

### 2.4. Intraoperative and postoperative findings

Intraoperative findings including the period from symptom onset to surgery and the surgical procedure were recorded. The operation time of unilateral and bilateral PH was documented. In addition, intraoperative complications were noted.

During hospitalization, the overall condition of each dog was evaluated. Postoperative complications including seroma, wound dehiscence, localized infection in the surgical wound, tenesmus, constipation, urinary incontinence and lameness were recorded. In addition, physical examination, CBC, serum chemistry, and abdominal radiography were performed during hospitalization, as needed.

After discharge, a physical examination, digital rectal examination, CBC, serum chemistry, and abdominal radiography were performed on every consultation day. Presence or absence of recurrence was also assessed. Follow-up consultations after discharge typically commenced approximately 2 weeks after surgery. Based on the patient’s condition, the intervals between subsequent consultation days were gradually extended. During these consultations, comprehensive assessments were conducted, including physical and rectal examinations. Additionally, radiography and blood tests were carried out as needed. Postoperative outcomes were determined as excellent (medical management was needed for minor complications, but PH-related clinical symptoms improved), good (PH-related clinical symptoms improved, but medical management was needed for major complications), and poor (medical management was needed for major complications, and PH-related clinical symptoms did not improve) based on information from medical records or telephone records from referring veterinarians or owners.

## 3. Results

### 3.1. Signalment

The results are summarized in Table 1. All dogs were male, of which 7 (32%) were neutered, and 15 (68%) were intact. The most common breeds were Miniature Dachshunds (10 cases, 45%), Chihuahuas and Pembroke Welsh Corgis (3 cases each, 14%), Beagle, Border Collie, French Bulldog, Papillon, Siberian Husky, and Toy Poodle (1 case each, 5%). The median age of the dogs was 10 years (range, 5–14 years), and the median weight was 6.8 kg (range, 2.3–20 kg).

**Table 1:** Summary of the dogs treated by herniorrhaphy with a cone-shaped polypropylene mesh.

No.	Age (y-o)	Breed	BW (kg)	Castration	Hernial side	Clinical signs			Operation time (mins)	Additional procedures	Abdominal skin incision	Prineal skin incision	Hospitalization period (days)	Follow-up period (days)	Postoperative prognosis	
						Defecation	Defecation assistance	Urination							Short-term	Long-term
1	5	Welsh Corgi Pembroke	14.5	None	Bilateral	Tenesmus	NA	NA	112	Colo + Cysto	Median	–	7	775	Excellent	Excellent
2	7	Siberian Husky	20.0	–	Bilateral	Tenesmus	Needed	NA	116	Colo + Cysto	Median	–	6	355	Excellent	Excellent
3	7	Beagle	14.0	None	Bilateral	Tenesmus	Needed	NA	163	Colo + Cysto	Median	Semicircular	11	754	Excellent	Poor (Recurrence)
4	8	Chihuahua	3.7	None	Bilateral	Tenesmus	Needed	NA	97	–	–	Semicircular	12	110	Good (Temporary lame)	Excellent
5	9	Miniature Dachshunds	5.0	–	Bilateral	Tenesmus	NA	NA	79	–	–	–	10	605	Excellent	Excellent
6	10	Miniature Dachshunds	7.0	None	Bilateral	Tenesmus	NA	NA	115	–	–	–	6	1,788	Excellent	Good (Laceration)
7	10	Miniature Dachshunds	6.0	None	Bilateral	Tenesmus	Needed	NA	96	Colo + Cysto	Median	-	8	1,512	Excellent	Excellent
8	10	Miniature Dachshunds	9.5	–	Bilateral	Tenesmus	Needed		85	–	–	–	8	3,067	Excellent	Excellent
9	11	French bulldog	12.6	–	Bilateral	Tenesmus	Needed	Hematuria	111	–	–	–	14	367	Excellent	Poor (Recurrence)
10	11	Miniature Dachshunds	5.1	None	Bilateral	Tenesmus	Needed	NA	123	Colo + Cysto	Median	–	6	2,765	Excellent	Excellent
11	11	Miniature Dachshunds	5.2	None	Bilateral	Tenesmus	NA	NA	144	Colo + Cysto	U-shaped	–	10	1,452	Excellent (Temporary seroma)	Excellent
12	12	Chihuahua	2.3	None	Bilateral	Tenesmus	NA	NA	91	–	–	–	9	893	Excellent	Excellent
13	12	Welsh Corgi Pembroke	11.2	None	Bilateral	Tenesmus	NA	NA	98	–	–	–	6	801	Excellent	Excellent
14	12	Miniature Dachshunds	4.8	None	Bilateral	Tenesmus	NA	NA	102	Colo + Cysto	Median	-	17	2,049	Excellent	Excellent
15	13	Miniature Dachshunds	6.5	None	Bilateral	Tenesmus	NA	NA	107	Colo	Median	–	9	989	Excellent	Poor (Contralateral PH)
16	14	Papillon	2.4	None	Bilateral	Tenesmus	Needed	NA	134	Colo + Cysto	Median	–	13	330	Excellent	Excellent
17	9	Miniature Dachshunds	4.7	None	Left	Tenesmus	Needed	NA	52	–	–	–	10	1,630	Excellent	Poor (Contralateral PH)
18	13	Miniature Dachshunds	8.7	None	Left	Tenesmus	Needed	NA	34	–	–	–	10	146	Excellent	Excellent
19	8	Border Collie	20.0	–	Right	Tenesmus	Needed	NA	62	–	–	–	6	1,198	Excellent	Excellent
20	8	Toy Poodle	8.6	None	Right	Tenesmus	NA	Incontinence	77	–	–	–	14	2,829	Excellent	Excellent
21	9	Chihuahua	3.9	–	Right	Tenesmus	Needed	NA	86	Cysto	Median	–	11	1,300	Excellent	Poor (Recurrence)
22	12	Welsh Corgi Pembroke	12.1	–	Right	Tenesmus	NA	NA	59	–	–	–	6	212	Excellent	Excellent

BW, Body weight; y-o, years old; Colo, colopexy; Cysto, cystopexy; POD, postoperative day; NA, no abnormalities. Outcome was determined as follows: Excellent- medical management was needed for minor complications but the PH-related clinical symptoms improved; Good-The PH-related clinical symptoms improved but medical management was needed for major complications; Poor- Medical management was needed for major complications and the PH-related clinical symptoms did not improve.

### 3.2. Clinical signs

Six dogs had unilateral PH (two on the left side and four on the right side), and 16 dogs had bilateral PH. PH-related clinical symptoms, including tenesmus, were observed in 22 dogs (100%), of which 12 dogs (55%) required the owner’s assistance because of difficulty in defecation. Until surgery, supportive treatment was provided by administering a fecal softener, such as lactulose, in eight cases (36%). Clinical signs related to urination were observed in two dogs (9%), including urinary incontinence in one dog (5%) and hematuria in one dog (5%) (Table 2).

**Table 2:** Summary of reccurrence and new onset casees after herniorrhaphy with the cone shaped polypropylen mesh.

No.		Age (y-o)	Breed	BW (kg)	First hernial side	Second hernial side	Symptoms	Recurrence date (POD)	Reoperation	Postoperative complications
3	Reccurence	7	Beagle	14.0	Bilateral	Right	Tenesmus	389	None	–
9	Reccurence	11	French bulldog	12.6	Bilateral	Right	Tenesmus	342	PM resuturing	None
21	Reccurence	9	Chihuahua	3.9	Right	Right	Swelling in the perineum	920	None	–
15	Contralateral PH	13	Miniature dachshund	6.5	Bilateral	Vetral	Tenesmus	989	None	–
17	Contralateral PH	9	Miniature dachshund	4.8	Left	Right	Tenesmus	76	PM implant	Recurrence (POD 1,630)

BW, Body weight; y-o, years old; POD, postoperative day; PM, Polypropylene mesh.

### 3.3. Intraoperative findings

The median period from symptom onset to surgery was 3 months (range, 0–61 months). Perineal herniorrhaphy using a cone-shaped PM was feasible in all dogs; only colopexy was performed in 1 dog, only cystopexy was performed in 1 dog, both were performed in 8 dogs, and neither was performed in 12 dogs. One dog required a preputial U-shaped skin incision in the foreskin, and two dogs required a semicircular incision at the ventral portion of the anus on the perineum. The median operation time was 60.5 min (range, 34–86 min) for unilateral PH and 109 min (range, 79–163 min) for bilateral PH. Intraoperative complications including bleeding, rectal perforation and cardiac arrest did not occur.

### 3.4. Postoperative progress

During hospitalization, perioperative fluid therapy and analgesics administration were continued in all dogs, and their general conditions improved postoperatively. In most cases, mild swelling at the surgical areas was observed a few days after surgery, but resolved before discharge. Importantly, none of the dogs developed any infections in the surgical wounds. Postoperative tenesmus was continued in 6 dogs (27%). All dogs were managed with commercial high fiber diet and medical management with lactulose (Nichi-Iko Pharmaceutical Co., Ltd., Toyama, Japan) and/or mosapride citrate hydrate (Pronamide®; DS Pharma Animal Health Co., Ltd., Osaka, Japan). No clinical signs related to urination were observed in any of the dogs. The median duration of hospitalization was 9.5 days (range, 6–17 days). An excellent prognosis was observed in 21 dogs (95%) and a good prognosis in 1 dog (5%) during the short-term (< 2 weeks) follow-up period. In Case 4, postoperative pain and was lameness in the left hind leg were showed next day after surgery, attributed to the sciatic nerve stimulation by the suture used during PH reconstruction. The sutures were removed on postoperative day (POD) 1, and the lameness disappeared 2 months later.

The median follow-up period at our hospital was 941 days (range, 110–3,067 days). Twenty-two dogs were available for long-term (> 2 weeks) postoperative evaluations. Excellent prognoses were observed in 16 dogs (73%), a good prognosis in 1 dog (5%), and poor prognoses in 5 dogs (23%) during the long-term follow-up period. Dogs with excellent prognoses had preoperative control over tenesmus, which had been observed before surgery, through the use of commercial high fiber diet and medical management. According to responses from the owners and referring veterinarians in the questionnaire, none of the dogs appeared to have been negatively affected by the surgery. The functional outcomes and quality of life remained high for all dogs.

The cases with poor prognoses included those with PH recurrence at the surgical site or those who developed contralateral PH during long-term follow-up. Recurrence was observed in three dogs (14%) on long-term POD. Case 3 had bilateral PH. The patient showed difficulty in defecation on POD 389, and recurrence was suspected. However, the difficulty in defecation improved after surgery, and the dog owner did not opt for revision surgery. Case 9 also had bilateral PH. The patient showed difficulty in defecation on POD 342 and developed PH on the right side. On POD 354, the PH was reconstructed on the right side. Postoperatively, clinical symptoms improved. Case 21 had right-sided PH. On POD 920, the swelling was observed in the right perineum; however, PH symptoms, including difficulty in defecation or urination, did not develop.

The development of contralateral PH was observed in two dogs (9%) during long-term follow-up. Case 15 had ventral PH. It was difficult to defecate on POD 989, and recurrence was suspected. Case 17 had left-sided PH. It showed difficulty in defecation on POD 76 and developed PH on the right. On POD 105, reconstruction of PH on the right side was performed. The clinical symptoms improved postoperatively. On POD 1,630, telephone records revealed no recurrence. Laceration occurred on the skin over the PM implantation site in one dog during long-term POD. Case 6 developed a skin laceration on POD 1,480. In the present case, no infection was observed; therefore, the skin was sutured. The patient had a good prognosis.

## 4. Discussion

It has been reported that the most commonly used technique, IOT, has no postoperative complications in 51.2 to 93% of the cases, with a recurrence rate of 0 to 27.4% (5, 6). Also, postoperative complications have been reported to occur in 21.4% of the cases treated with semitendinosus muscle transposition, which can be used even for serious cases, and recurrence of PH occurs in 14.3% of the cases (7). In our study, 32% of the cases had major complications, and 14% had a recurrence of PH, which is comparable with the findings of other studies. In the long-term follow-up period, 73% of the patients had an excellent prognosis. These findings suggest that PH reconstructive surgery using a cone-shaped PM is a useful alternative to PH reconstructive surgery in dogs.

Muscle transposition is the general surgical procedure used for PH repair, with IOT being the most commonly used technique and often used in combination with other techniques (11, 16, 18). However, IOT is difficult to perform in cases of postoperative recurrence of PH (5). Similarly, surgical techniques using autologous tissues, including IOT, may be difficult in cases with severe atrophy of the pelvic diaphragm muscles. Conversely, semitendinosus muscle transposition tends not to be affected by muscle atrophy in PH; hence, this technique may be indicated for cases with muscle atrophy (7, 19). However, semitendinosus muscle transposition involves a wide area of transposed muscle, which poses a high risk of postoperative seroma (19). In contrast, the use of artificial material in our study eliminated the need for muscle transposition; thus, this technique could be applied to cases with severe muscle atrophy and can also minimize surgical wounds.

A previous study utilized a PM to reinforce the muscle transposition for PH reconstruction (16). In this approach, an IOT surgery was initially performed, and then a flat PM sheet was placed over the transposed internal obturator and external anal sphincter muscles, securing it with sutures. The recurrence rate of this method was 12.5% (16), The rate was comparable to the previous study using IOT alone (5, 6). Consequently, the effectiveness of using a sheet PM as the reinforcement of PH reconstruction remains uncertain. Another technique, without the combination of IOT surgery, involved closing PH using a flat PM sheet alone. This approach resulted in a recurrence rate of 0%, although the postoperative follow-up was only 51.5 days (range, 39–65 days) (15). Therefore, whether the pelvic septum repaired with a flat PM can maintain its strength over an extended period remains unclear. In our study, the cone-shaped PM not only successfully closed the hernia but also prevented rectal deviation and caudal shifting of anus over an extended period. Moreover, the cone-shaped design could adapt to various hernial sizes and shapes. Therefore, the cone-shaped PM appears to provide a logical approach to PH reconstruction. Nevertheless, further investigations are required to determine the most suitable PM design for canine PH repair.

In a previous study, 3 of the 21 dogs treated for unilateral PH developed contralateral PH (5). It has also been reported that there have been no cases of recurrence after bilateral surgery for unilateral PH for more than 2 years (6). In our study, the cases occurred contralateral PH was observed in 2 of the 22 dogs during long-term POD. This is because our method can reconstruct the pelvic septum well but distributes the abdominal pressure to one side. In the future, it will be necessary to establish preoperative diagnostic methods for patients at high risk of contralateral PH and manage new-onset, high-risk cases of PH.

A previous study showed that good PH surgical outcomes could be achieved by suturing the sacrotuberous ligament during PH reconstruction (9). However, suturing to the sacrotuberous ligament can cause paralysis of the hind legs (20, 21). The PH was repaired by suturing the cone-shaped PM to the sacrotuberous ligament. In only one case, lameness of the hind legs was observed, which was thought to be caused by the involvement of the sciatic nerve when suturing the sacrotuberous ligament. The lameness was improved by removing the suture and resuturing the PM on POD 1. Although the incidence of sciatic nerve injury is low, this study demonstrates that this technique poses a risk of sciatic nerve injury.

Major complications of PM implantation procedures have been reported to include surgical wound dehiscence (12.5%) and sepsis due to infection (5.6%) (16). Prognosis tended to be poor in cases with suspected infection due to implantation of the PM. PM implantation has also been reported to pose a risk of foreign body reactions (22). In our study, there were no foreign body reactions to the PM or infections after reconstruction. However, laceration of the skin over the PM implantation site was observed during the long-term postoperative follow-up. This phenomenon was thought to be caused by thinning of the skin and its surrounding tissues after PM implantation, owing to friction between the PM and skin. One patient had skin dehiscence requiring skin closure treatment. Therefore, this long-term complication is suggested to potentially occur after cone-shaped PM implantation; therefore, owners should be informed of the risk of skin laceration.

In our study, colopexy or cystopexy was performed in 10 cases to prevent the caudal displacement of the abdominal organs. A previous report demonstrated no significant association between colopexy/cystopexy and postoperative complications, and that these procedures did not affect the long-term prognosis of PH (23). In our study, usefulness of these procedures was not yet determined in the postoperative course. However, caudal displacement of the tissues including anus, rectum, bladder and prostate can complicate PH reconstruction surgery. Our findings suggest that performing colopexy and cystopexy may facilitate the suturing of the cone-shaped PM to the pelvic diaphragm (sacrotuberous ligament, internal obturator muscle, and external anal sphincter muscle). Therefore, colopexy and cystopexy are thought to be effective for safe and secure PH reconstruction with the PM when there is caudal displacement of anus, rectum, bladder and prostate. Nevertheless, further investigations are required to determine the effects of colopexy and cystopexy on the postoperative course.

One of the major advantages of the Trendelenburg position used in our study is that it enables the sequential performance of castration, colopexy, cystopexy and PH reconstruction without repositioning the patient. A previous study has demonstrated that caudal scrotal castration is feasible in the prone position when carrying out the PH reconstruction (24). However, colopexy and/or cyctopexy can not be concurrently performed without repositioning the patient. In addition, a previous study indicated that the supine position with the hind legs held cranially might reduce the volume of abdominal organs prolapsed into the PH area during reconstruction surgery compared to the prone position (25). In our study, PH reconstruction surgery was carried out in the Trendelenburg position, which the patients were held in a supine position and the head lowered by tilting the operating table. Therefore, the Trendelenburg position might induce a more cranial shift of the abdominal organs compared to the supine position. A previous study on PH reconstruction in the Trendelenburg position reported that it facilitates the reduction in the volume of abdominal organs prolapsed into the PH area during surgery (12). These findings suggest that the Trendelenburg position is beneficial for PH reconstruction. Further clinical studies are required to determine the more suitable patient position for PH reconstruction in dogs.

The major limitations of our study included the small number of cases and inconsistency in the follow-up period for treated cases. It is necessary to treat more cases of PH using PM and conduct long-term follow-ups (2 or more years) to investigate possible postoperative complications. This technique poses the risk of foreign body reaction as a postoperative complication, which makes it difficult to perform in patients with autoimmune diseases (16, 22).

In conclusion, our study suggests that PH reconstruction surgery using a cone-shaped PM may be a viable treatment method for PH in dogs. Therefore, a cone-shaped PM could serve as an alternative treatment option for canine PH reconstruction.

## Data availability statement

The original contributions presented in the study are included in the article/supplementary material, further inquiries can be directed to the corresponding author.

## Ethics statement

The animal studies were approved by Ethical Committee of Nihon University Animal Medical Center. The studies were conducted in accordance with the local legislation and institutional requirements. Written informed consent was obtained from the owners for the participation of their animals in this study.

## Author contributions

TH: Conceptualization, Data curation, Formal analysis, Investigation, Methodology, Visualization, Writing – original draft, Writing – review & editing. KuI: Conceptualization, Data curation, Formal analysis, Investigation, Methodology, Supervision, Visualization, Writing – original draft, Writing – review & editing. MS: Data curation, Investigation, Methodology, Supervision, Writing – original draft, Writing – review & editing. KT: Data curation, Investigation, Methodology, Supervision, Writing – original draft, Writing – review & editing. OY: Data curation, Investigation, Methodology, Writing – original draft, Writing – review & editing. KaI: Data curation, Investigation, Methodology, Writing – original draft, Writing – review & editing. RT: Data curation, Investigation, Methodology, Writing – original draft, Writing – review & editing. KA: Data curation, Formal analysis, Investigation, Methodology, Project administration, Supervision, Visualization, Writing – original draft, Writing – review & editing.
